# Trends in health behavior at an afterschool program: the impact of COVID-19 on students’ behavior

**DOI:** 10.21203/rs.3.rs-2679660/v1

**Published:** 2023-03-28

**Authors:** Emily Yamron, Jee-Young Moon, Paul Meissner, Judith Wylie-Rosett, Viswanathan Shankar, Jessica Rieder

**Affiliations:** Albert Einstein College of Medicine; Albert Einstein College of Medicine; Montefiore Medical Center; Albert Einstein College of Medicine; Albert Einstein College of Medicine; Children’s Hospital at Montefiore

**Keywords:** childhood obesity, afterschool program, physical activity, COVID-19

## Abstract

**Background:**

A minority of American youth meet CDC physical activity (PA) recommendations; children in the Bronx face additional structural barriers to engaging in PA. The B’N Fit Power expansion draws on pilot programming to increase the proportion of middle school students who engage in one hour of daily PA. The COVID-19 pandemic presented additional obstacles, including increased food insecurity and suspension of organized PA programming. This research aimed to evaluate differences in baseline target behavior attainment before and after the start of the COVID-19 pandemic to inform future programming to help children reduce their risk of obesity.

**Methods:**

Afterschool program leaders at three Bronx public schools collected demographic and target behavior data at baseline and attendance data throughout the school year.

**Results:**

During the 2016–2017 and 2017–2018 school years, 76 students enrolled and completed one year of programming, which was administered at a single site (61 % Hispanic, 46% female). Of these, 76 (100%) completed a baseline target behaviors questionnaire. During the 2021–2022 school year, 417 students enrolled and completed one year of programming at one of the three sites (70% Hispanic, 48% female). 89 (21%) completed a baseline target behaviors questionnaire. Participants surveyed after the start of the COVID-19 pandemic reported drinking more sugar-sweetened beverages (Median=3 daily, IQR 2–5), sleeping less (Median=8 hours daily, IQR 6–9 hours), and consuming fast food more frequently (Median=1 time weekly, IQR 0 times weekly-2 to 3 times weekly) than those surveyed prior to the start of the pandemic. The number of PA hours completed each week trended toward significant decline (Median=3, IQR 2–5, p=0.09) in students tracked after the start of the pandemic.

**Conclusions:**

The attainment of several target behaviors among school children linked to the reduction of childhood obesity declined during the COVID-19 pandemic. These findings can be applied to enhancing existing real-world afterschool PA programming.

## Background

A minority of American youth meet CDC physical activity recommendations (1). Nearly one in five children in the United States has obesity; these rates are higher among Hispanic and non-Hispanic Black children (2). Obesity may contribute to as well as be the result of low physical activity. Under 1/4 of youth meet physical activity guidelines (1). While the long-term outcomes of the COVID-19 pandemic are still being evaluated, research suggests that children worldwide engaged in less organized physical activity during the pandemic (3).

Children and adolescents in urban settings such as the Bronx face additional barriers to engaging in physical activity. The Bronx has the state of New York’s highest proportion of children living in poverty (38% immediately before the start of the COVID-19 pandemic) and is a majority-minority county (4). Nearly 1/3 of Bronx households experience severe housing cost burdens (4). Bronx schools have high absentee and low completion rates (68% before the start of the COVID-19 pandemic) (4). The COVID-19 pandemic has exacerbated existing socioeconomic and health disparities. The Bronx unemployment rate in 2022 was the state’s highest at 16%, and the Bronx experienced the highest per capita death rate of New York’s 62 counties (5). The low socioeconomic status of Bronx teens limits access to recreational resources and physical activity opportunities that may enrich their lives and reduce health disparities (6). Among Bronx 6th graders, 31% participated in physical activity 1–3 days per week, 30% participated 4–6 days, and 34% participated for all seven days. Almost 45% of 6th and 7th graders watched three or more hours of TV, videos, or play on cell phones, iPads, or other tablets or handheld video games daily. More Bronx 6th graders drank one or more sugary drinks daily than their counterparts in other boroughs (78% vs. 35%) (7). Rates of obesity prevalence are higher for Bronx youth of Hispanic and Black background (22.3%) than non-Hispanic white youth (8).

The B’N Fit Power pilot study aimed to address high youth obesity prevalence in the Bronx by evaluating the real-world efficacy of a multipronged intervention to increase physical activity and nutrition activities in afterschool programming. B’N Fit Power was piloted at one public middle school in the Bronx in 2016; the programming and its outcomes have been detailed previously (9).

The B’N Fit Power expansion to two additional sites draws on lessons learned from the pilot afterschool physical activity and nutrition programming to increase the proportion of middle school students who achieve seven core nutrition and physical activity behaviors. This research aims to evaluate differences in baseline target behavior attainment before and after expansion and the COVID-19 pandemic shutdown to inform future programming to help children reduce their risk of obesity.

## Methods

### Study design

The study uses a repeated cross-sectional design to examine the impact of the B’N Fit Power afterschool program on target behavior attainment. The B’N Fit Power program was piloted in one public middle school in the Bronx during 2016–2018 and expanded to two additional schools in the Bronx in 2021 in a real-world setting. Students’ target behaviors and afterschool attendance were collected 3–4 times during the school year. In this study, we aimed to compare baseline target behavior outcomes and attendance throughout the school year before and after the start of the COVID-19 outbreak. The Albert Einstein College of Medicine IRB approved the pilot (IRB #2015–5917) and the expansion study (IRB #202113679). For the pilot study, students were directly involved and informed consent was obtained from parents. For the expansion study, students were not directly involved and the Albert Einstein College of Medicine IRB waived written informed consent.

### Setting

The B’N Fit Power was implemented at three public middle schools in the Bronx, New York.

### Program

B’N Fit Power involves the integration of daily physical activity components into a community-run afterschool program that takes place in public schools in the Bronx. A detailed description of the B’N Fit Power program is described elsewhere (9). As the programming expanded from one school to three, monthly meetings with the community center staff administering the program ensured that every site had consistent opportunities for students to participate in physical activity.

In expanding from one site to three sites, staff members from the overseeing community organization met monthly with site youth directors to ensure that PA programming was consistently implemented across sites, discuss challenges in tracking, and troubleshoot problems.

### Student enrollment

All students participating in afterschool programming at the three schools received B’N Fit Power programming. This is a change from the pilot pre-COVID-19 cohort, wherein a subset of students participating in the afterschool program was specifically screened, consented, and enrolled in the study/program, as previously described (9). When programming resumed after the COVID-19 shutdown, all data used in the study were already being routinely collected for non-research purposes. Thus, the waiver of informed consent and HIPAA authorization was approved by the Montefiore/Einstein Institutional Review Board, and no additional research consent was required.

### Data collection

The schools where programming took place provided data on student demographics, attendance at afterschool physical activity programming, and target behaviors. Outcome measures consisted of data collected from routine afterschool programming by youth program leaders at each site. Activities that constituted any physical activity type were coded as PA activities; the total number of hours students attended over the year was summed for each student. Target behaviors were measured by a survey administered routinely to students and included: how many days each week students ate breakfast, how many each week students ate lunch, how many servings of fruit students ate each day, how many servings of vegetable students ate each day, how many cups of unsweetened beverages students drank each day, how many cups of sweetened beverages students drank each day, how many hours of sleep students got nightly, how many times weekly students consumed fast food, how many times weekly students consumed unhealthy snacks, how many hours a day students engaged in physical activity, and how many hours of recreational screen time a student had each day. The student’s responses provided when surveyed at the start of the school year (September-October) were considered their baseline responses.

### Statistical analysis

Data analysis was conducted on data provided by schools. The number of PA hours was extracted from attendance data for activities relating to PA. Target behavior data was collected by administering a self-reported target behaviors survey directly to afterschool program participants.

The distribution of participant characteristics was summarized using descriptive statistics. The categorical variables were summarized using frequency count and percentages, while continuous scale variables were summarized as mean (standard deviation) or median (interquartile range) as appropriate.

Survey responses to target behavior were dichotomized as attained/not attained; “attainment” was aligned with evidence-based recommendations for each behavior. One point was allocated for each behavior attained. The points were then summed into a multi-component composite score with a maximum of seven; students received one point each for: (1) eating breakfast and lunch daily, (2) eating 2–3 servings of fruit daily, (3) eating 3–6 servings of vegetables daily, (4) drinking at least 8 cups of water daily and drinking no more than one cup of a sweetened beverage daily, (5) getting at least eight hours of sleep daily, (6) limiting fast food and unhealthy snack consumption to no more than once weekly, and (7) getting at least an hour of physical activity daily. An eight-component score was also computed; students could receive an additional point for having 2 hours a day or fewer of recreational screen time. Differences between groups pre- and post-shutdown cohorts were evaluated using the Mann-Whitney U-test for continuous or ordinal variables and Fisher’s exact test for categorical variables. Data were analyzed in R 4.2.0.

## Results

Table 1 summarizes students’ demographic information before (N=76) and after (N=417) the start of the COVID-19 pandemic shutdown. Before the beginning of the COVID-19 pandemic, 46% of students were female, and 62% were Hispanic or Latino. Among students who enrolled after the COVID-19 pandemic shutdown, 48% were female, and 70% were Hispanic or Latino.

Table 2 summarizes program attendance in general, physical activity programming, and target behavior attainment. For pre-pandemic attendance data, the median number of total hours of programming attended over the school year was 324 (IQR: 324–324); for post-pandemic shutdown attendance data, this declined to 99 (99–147, p<0.001). For pre-pandemic PA attendance data, the median number of PA hours attended over the school year programming through B’N Fit Power was 63.8 hours (IQR 40.5–92.2). For post-pandemic shutdown PA attendance data, the median number of PA hours attended over the school year (September-June) programming was much lower, at 20.5 hours (IQR 12–39.2, p<0.001).

Pre- and post-pandemic shutdown, the median baseline self-reported seven-component composite scores were 2 (IQR 2–3) and 2 (IQR=1–3, p=0.003), respectively. While the median did not change, the overall distribution shifted towards fewer target behaviors attained after the COVID-19 pandemic shutdown. When evaluating the eight-component score, which also included recreational screen time, the median pre-pandemic score was 3 (IQR=2–3), while the post-shutdown score was 2 (IQR=1–3, p=0.001).

**Figure 1:**
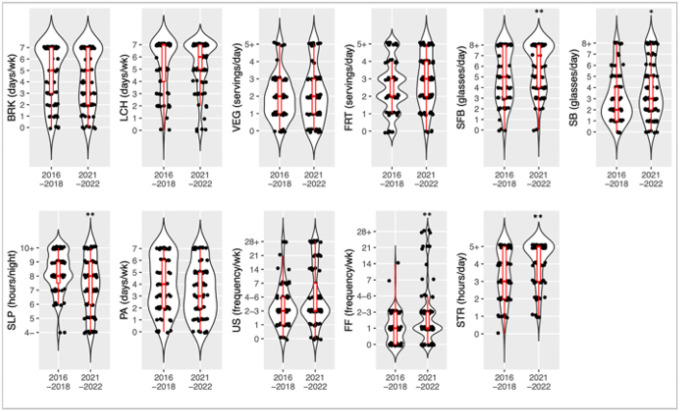
Distribution of target behavior responses BRK = number of days student consumed breakfast weekly LCH = number of days student consumed lunch weekly VEG = number of servings of vegetables the student consumed daily FRT = number of servings of fruit the student consumed daily SFB = number of cups of sugar-free beverages the student consumed daily SB = number of cups of sweetened beverages the student consumed daily SLP = number of nightly hours of sleep the student received PA = number of days the student did one hour of physical activity US = number of times weekly students consumed unhealthy snacks FF = number of times weekly students consumed fast food STR = number of daily hours spent on recreational screen time * = p-value 0.05 ** = p-value 0.01

Differences in individual pre-and post-pandemic shutdown behaviors at the beginning of the school year were also evaluated. Consumption of unsweetened beverages at baseline rose from 5 (IQR=3–8) to 7 (IQR 4–8, p=0.009) pre- to post-shutdown. Consumption of sweetened beverages at baseline also increased from 2 (IQR 1–4) to 3 (IQR 2–5, p=0.029). There were additionally statistically significant changes in the distribution number of hours of sleep and the frequency of fast-food consumption. The number of hours of daily screen time students engaged in rose significantly from 3 (IQR 2–5) to 5 (IQR 3–5, p=0.0005) The number of self-reported days in which students engaged in an hour of PA trended toward a significant decline from a pre-pandemic of 4 days (IQR 3.5–5) to 3 (IQR 2–5, p =0.090). There were no other significant changes between pre- and post-COVID-19 shutdown behaviors. The distribution of target behavior frequency can be seen in figure 1.

**Figure 2:**
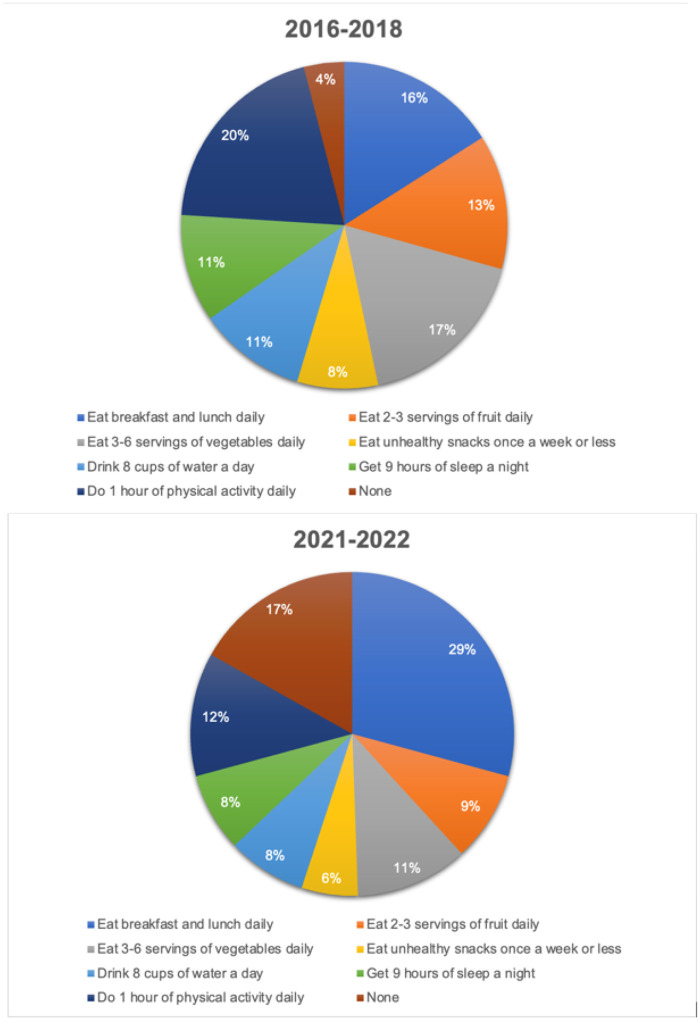
Goal target behaviors

In the pre-pandemic period, 20% of students identified getting one hour of physical activity as their target behavior goal; post-pandemic shutdown, 12% identified this as their goal. Pre-pandemic, 16% of students identified eating breakfast and lunch daily as their target behavior goal; post-shutdown, 29% identified this as their goal (figure 2).

## Discussion

The B’N Fit Power program has expanded from one NYC public school in the Bronx to three during the COVID-19 pandemic, allowing for greater reach among students. During the COVID-19 pandemic, afterschool program attendance records indicate a significant decline in PA hours completed during programming. Self-reported baseline target behavior attainment fell during the COVID-19 pandemic, with median composite scores declining in the post-pandemic cohort compared to the pre-pandemic cohort. Additionally, the proportion of students identifying physical activity as the target behavior they wanted to improve fell by nearly half. In contrast, the proportion who identified eating daily breakfast and lunch as their goal nearly doubled.

It was noted in the pilot of B’N Fit POWER that the targets of behaviors, sorted into binary “achieved/not achieved,” might obscure sub-threshold improvements in behavior; for example, going from no servings of vegetables daily to one (9). As noted in the pilot study, target behaviors align with evidence-based recommendations for pediatric nutrition and physical activity behaviors (10, 11, 12, 13); however, even in the original pilot study, improvements were seen that did not achieve the recommended level. In this analysis, we examined both binary achievements of target behaviors through the composite score and the pre-/post-pandemic shutdown distributions to look at sub-threshold changes in behavior (e.g., an increase in PA hours from 0 to 4 weekly, which does not meet the target of 7 hours weekly). Given that composite target behavior scores were low, examining trends in individual target behaviors was necessary. Specifically, it was essential to see if participants’ behaviors were changing to the degree that was insufficient to change their status on the binary score yet may have demonstrated either positive or negative underlying behavioral changes. When examining this data, post-pandemic shutdown students appeared to drink more water and sweetened beverages, sleep less, engage in more screen time, and consume fast food more frequently. Additionally, a decline in the number of days participants had an hour of physical activity had a trend toward a significant decrease, from four to three days weekly. While consumption of fruits, vegetables, and breakfast/lunch at baseline was lower than the goal, no significant pre/post-pandemic shutdown differences in behavior were observed.

The rise in childhood inactivity during the COVID-19 pandemic has been previously described in the literature (3). Regular physical activity and healthy diet behaviors are not only associated with obesity prevention and management in youth but are also critical for maintaining health into adulthood (14). This data reveals that several behaviors were negatively impacted by the pandemic, including increased utilization of screen time recreationally, increased consumption of fast food and sweetened beverages, and decreased sleep. Additionally, a decline in the number of days in which participants engaged in one hour of daily PA participation trended toward significance.

This study’s backslides in target behavior attainment underscore the importance of building healthy behaviors in children’s environment, including their school and afterschool programming. During this critical period of regression, participation in PA programming has fallen. This could be due to various factors, including adjusting PA options to be COVID-safe, interruptions in attendance due to personal illness, and scaling back programming compared to pre-pandemic levels. It is vital to continue to provide robust PA programming to stem any backslide in daily PA participation. While meeting daily physical activity recommendations is important in its own right, data show that this goal can also improve other behaviors, such as sleep and screen time (15, 16).

Pre-pandemic, the target behavior that a significant proportion of students was interested in working toward was participating in one hour of daily physical activity; post-pandemic shutdown, the target behavior with the most interest was consuming daily breakfast and lunch. However, there was no significant shift in the median number of days students consumed these meals. This observed discrepancy between the desired to consume daily breakfast and lunch yet no baseline change in this behavior may reflect increasing food insecurity; there was a nearly one-third increase in food insecurity during the COVID-19 pandemic (17). Food insecurity and obesity are often coexisting phenomena in low-socioeconomic communities (18); it is essential that programming be conscious of this. Alternatively, it is possible that students selected this goal because it seemed attainable due to changes in school lunch programming; since 2017, NYC public schools have offered universal free school lunches (19). The students who participated in the B’N Fit pilot selected a goal at baseline before the implementation of universal free school lunch; increased access for students taking the survey in the 2021–2022 school year may have influenced their choice.

One limitation of this research is the differential completion of baseline target behavior surveys pre- and post-pandemic shutdown. All students participating in the pilot program completed the target behaviors survey. However, during the program expansion in the 2021–22 school year, all students participating in afterschool programming received the B’N Fit Power program. While this increased the program’s reach, staffing limitations prevented all students from taking the baseline behaviors survey; consequently, the survey response rate was lower in the expansion than in the pilot. However, this analysis broadly shows that the COVID-19 pandemic has had an unmistakable impact on behaviors linked to childhood obesity.

Data from this real-world program provide insight into how specific obesogenic behaviors can be intervened upon in future programming to help children engage in healthier lifestyle behaviors and reduce their risk of obesity. The B’N Fit Power expansion offers an opportunity to do just this.

This research can inform future programming by further investigating participant desires and aligning programming to them. If, as hypothesized, the shift to identifying daily breakfast and lunch as a target goal reflects increased food insecurity, then expanding programming that supports food security will likely be beneficial. For example, the sites are currently working to begin hydroponics garden towers; these towers will provide fresh produce to participants, potentially promoting vegetable consumption and reducing burdens created by food insecurity.

## Conclusion

Overall, this study found that target behavior attainment for behaviors related to obesity prevention in youth declined after the start of the COVID-19 pandemic. Consumption of sugar-sweetened beverages and fast food increased, while hours of sleep declined; hours of physical activity programming also fell after the pandemic. As programming resumes and expands to additional sites, these insights can increase the number of physical activity hours offered, provide education related to healthy nutrition and sleep habits, and tailor programming to specific student needs.

## Figures and Tables

**Table 1,:** Demographic information

School	School 1		School 2	School 3
	2016–18	2021–22	2021–22	2021–22
Number of students enrolled in afterschool programming	76	182	96	139
Age, years (SD)	12.4 (1.0)	12.2 (1.0)	12.0 (0.8)	11.8 (1.1)
Gender				
Male	41 (53.9)	97 (53.3)	49 (51.0)	71 (51.1)
Female	35 (46.1)	85 (46.7)	47 (49.0)	67 (48.2)
Neither Male nor Female	0 (0)	0 (0)	0 (0)	1 (0.7)
Race and Ethnicity				
Hispanic or Latinx	47 (61.8)	133 (73.1)	42 (43.8)	117 (84.1)
Black, Not Hispanic or Latinx	19 (25)	42 (23.1)	12 (12.5)	11 (7.9)
Other, Not Hispanic or Latinx	10 (13.2)	7 (3.8)	42 (43.8)	11 (7.9)
Student completing baseline target behaviors survey	76 (100)	34 (18.6)	15 (15.6)	40 (28.8)

**Table 2,:** Comparison of baseline target behavior attainment and PA hours completed

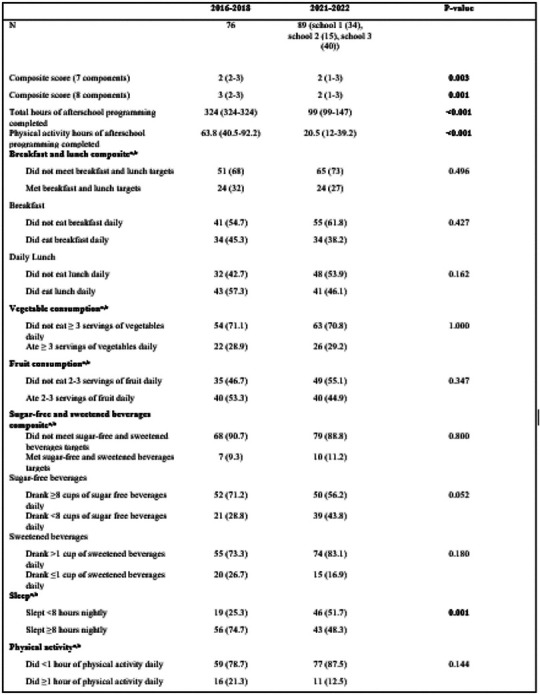

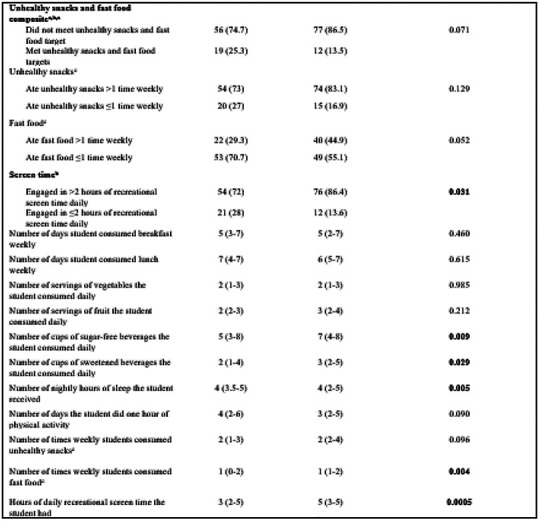

Values are median (IQR) for continuous or ordinal variables and count (%) for categorical variables. p-value by Mann-Whitney U-test for continuous or ordinal variables and by Fisher’s exact test for categorical variables

a Included in the 7-behavior composite scare

b Included m the S-behavior composite score

c The scale for fast food an unhealthy snack consumption was: 0= 0 times in the past week, 1= 1 tune in the past week. 2= 2–3 times in the past week. 3= 4–6 in the past week. 4= 1 time a day. 5= 2 tunes a day. 6= 3 tunes a day. 7= 4 tunes a day or more

## Data Availability

The datasets generated during and/or analyzed during the current study are not publicly available. Questions about access can be directed to the study PI, Dr. Jessica Rieder (H17MC29435, H17MC33892, and H17MC40191.

## Abbreviations

B’N Fit: Bronx Nutrition and Fitness Initiative for Teens
HIPAA: Health Insurance Portability and Accountability Act of 1996
BRK: number of days student consumed breakfast weekly
LCH: number of days student consumed lunch weekly
VEG: number of servings of vegetables the student consumed daily
FRT: number of servings of fruit the student consumed daily
SFB: number of cups of sugar-free beverages the student consumed daily
SB: number of cups of sweetened beverages the student consumed daily
SLP: number of nightly hours of sleep the student received
PA: number of days the student did one hour of physical activity
US: number of times weekly students consumed unhealthy snacks
FF: number of times weekly students consumed fast food
STR: number of daily hours spent on recreational screen time
